# Editorial: Methods and applications in molecular diagnostics

**DOI:** 10.3389/fmolb.2023.1239005

**Published:** 2023-07-17

**Authors:** Naina Arora, Anubha Chaudhary, Amit Prasad

**Affiliations:** School of Biosciences and Bioengineering, Indian Institute of Technology Mandi, Mandi, Himachal Pradesh, India

**Keywords:** diagnosis, molecular biology, PCR, NGS—next generation sequencing, cancer

The field of molecular diagnostics is experiencing a remarkable revolution, propelling us into an era of unparalleled advancements that are reshaping our understanding of diseases and revolutionizing healthcare. With every passing day, breakthroughs in molecular diagnostics are pushing the boundaries of our ability to detect, diagnose, and treat a vast array of medical conditions. In this captivating collection, we present a series of articles that serve as a testament to the astonishing progress made in this field across various disciplines, encompassing infectious diseases, cancer, and genetic disorders. These articles provide a window into the latest techniques, methodologies, and discoveries that are transforming the landscape of molecular diagnostics and opening new horizons for improved patient care and outcomes.

**FIGURE 1:**
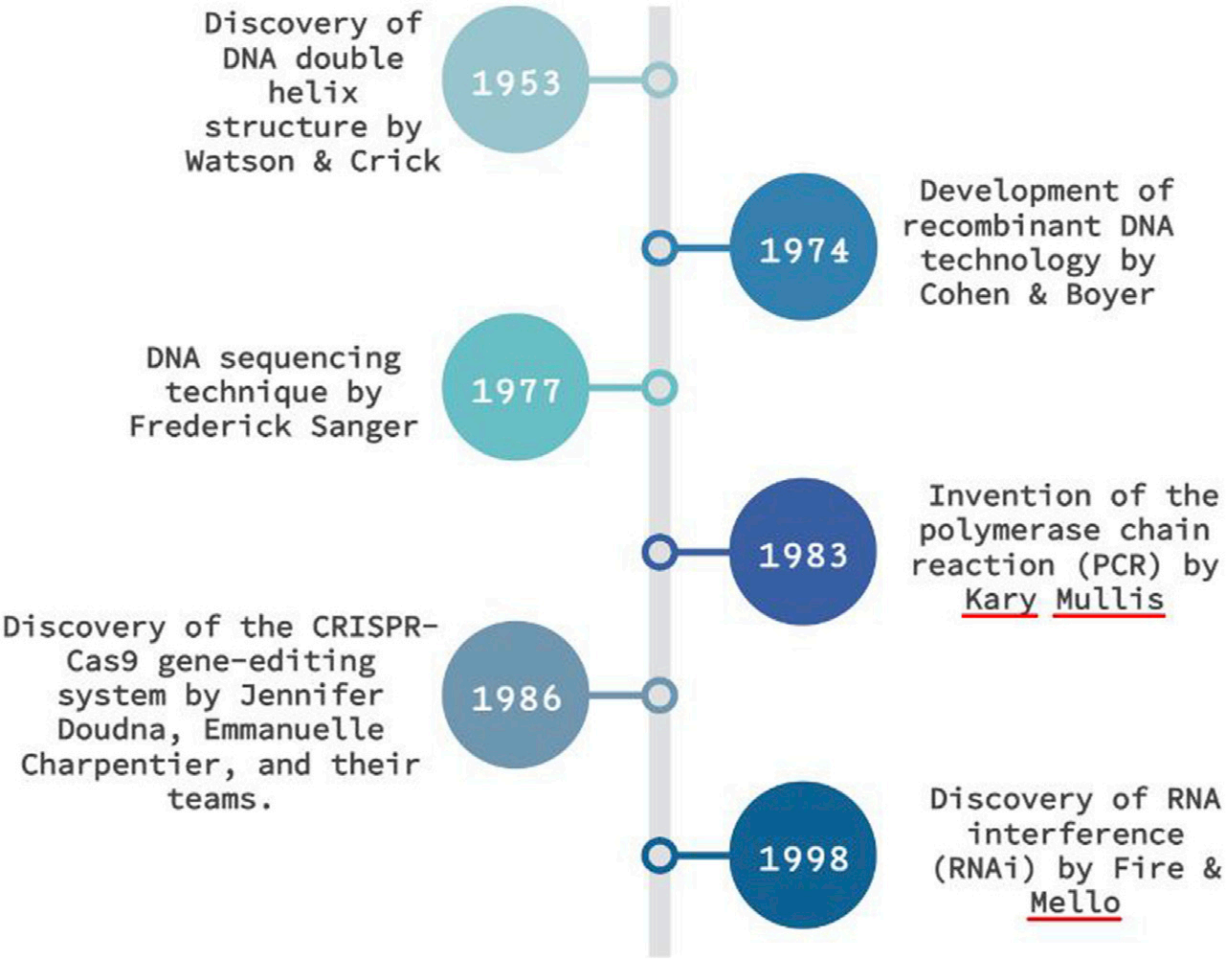
Milestones of molecular diagnosis techniques.

The journey of molecular diagnostics can be traced back to the ground-breaking discovery of the DNA double helix structure by Watson and Crick in the 1950s (Figure 1). This pivotal revelation laid the groundwork for transformative techniques, notably the invention of polymerase chain reaction (PCR) by Kary Mullis in 1983. PCR ushered in a new era in molecular diagnostics by enabling the amplification of minuscule DNA sequences that were previously undetectable. With its remarkable sensitivity, PCR has played a crucial role in exploring mutations and diagnosing various diseases. “Detection of CCR5Δ32 Mutant Alleles in Heterogeneous Cell Mixtures Using Droplet Digital PCR,” (Sorokina et al.) delves into the importance of detecting and quantifying mutant alleles in heterogeneous cell mixtures, particularly in the context of HIV research. The authors presented a new methodology that utilizes multiplex droplet digital polymerase chain reaction (ddPCR) to accurately quantify the presence of cells carrying the CCR5Δ32 mutation. This approach enables swift and precise measurement of mutant CCR5Δ32 alleles in cell mixtures, even at low levels as low as 0.8%. The sensitivity and potential of droplet digital PCR demonstrated in this study hold promise for application in other disease settings.

Building upon the foundation laid by PCR, DNA sequencing techniques pioneered by Sanger introduced a new paradigm of precision by facilitating the determination of precise DNA sequences. This breakthrough has propelled the field of molecular diagnostics forward, enabling comprehensive analyses of inherited disorders, identification of disease-associated genetic variations, and the emergence of personalized medicine based on an individual’s unique genetic profile. By enabling the determination of precise DNA sequences, these breakthroughs have propelled the field of molecular diagnostics forward, revolutionizing cancer research and patient care. Fuelled by the power of Next-Generation Sequencing (NGS) technology, DNA sequencing has become a cornerstone of molecular diagnostics in cancer research. This had enabled the rapid and cost-effective sequencing of millions of DNA fragments simultaneously, providing comprehensive genomic information with unprecedented depth and accuracy. This technological breakthrough has significantly accelerated our understanding of the genetic landscape of cancer, shedding light on the complex genomic architecture of tumours. The small RNA molecules and non-coding transcripts have been found to play crucial roles in regulating gene expression and influencing various cellular processes, including tumorigenesis. By studying the expression patterns and functional roles of miRNAs and lncRNAs, researchers have identified specific signatures and dysregulated molecules that contribute to tumour development and progression. For instance, the study “Classification of Hepatocellular Carcinoma Based on N6-Methylandenosine–Related lncRNAs Profiling” (Yin et al.) focuses on hepatocellular carcinoma (HCC), which is one of the most common types of malignancies worldwide. The authors performed a comprehensive analysis of 186 m6a-related lncRNAs to identify clusters associated with overall prognosis in HCC. They developed prognostic signatures based on four m6A-related lncRNAs that could predict the overall survival of HCC patients. This study highlights the power of exploring lncRNAs in cancer classification and prediction. Additionally, the study “Development of next-generation sequencing (NGS) has provided useful genetic information to redefine diagnostic, prognostic, and therapeutic strategies for the management of acute leukemia (AL)” (Vicente-Garcés et al.) demonstrates the utility of targeted NGS panels in pediatric acute leukemia. The authors validated the AmpliSeq™ for Illumina® Childhood Cancer Panel, which includes common genes associated with childhood cancer, and assessed its feasibility in routine diagnostics. The panel showed high sensitivity and specificity for detecting genetic variants and fusion genes, and its clinical utility was demonstrated in refining diagnosis and identifying targetable mutations. This study highlights the application of NGS in improving diagnostic accuracy and guiding therapeutic decisions in pediatric cancer.

By integrating the insights gained from genomic profiling, miRNA signatures, and dysregulated lncRNAs, molecular diagnostics has the potential to revolutionize cancer diagnosis, treatment selection, and patient monitoring. The comprehensive genomic information obtained through NGS, including the analysis of miRNAs and lncRNAs, provides unprecedented opportunities for personalized medicine. This approach enables the identification of specific miRNA or lncRNA targets for therapeutic intervention, allowing for tailored treatment strategies based on the unique molecular characteristics of each patient’s tumour.

These advancements in molecular diagnostics have paved the way for the identification and quantification of biomarkers, which are measurable indicators of biological processes and conditions. Real-time PCR and microarray analysis have played pivotal roles in the detection and quantification of specific biomarkers. These innovations have revolutionized disease diagnosis, prognosis, and treatment monitoring, empowering clinicians to make informed decisions regarding targeted therapies and optimizing treatment outcomes. Moreover, molecular diagnostics has brought about a transformative shift in the detection and surveillance of infectious diseases. Traditional methods, such as culturing and microscopy, often suffer from limited sensitivity and time-consuming procedures. In contrast, molecular techniques, exemplified by nucleic acid amplification tests (NAATs) like PCR, have emerged as powerful tools capable of rapid and accurate pathogen identification. This capability facilitates early diagnosis, leading to enhanced patient care and effective epidemiological surveillance for prompt outbreak investigations and monitoring of antimicrobial resistance, thus safeguarding public health on a global scale. Molecular diagnostics has witnessed the emergence of a remarkable innovation known as point-of-care testing (POCT), revolutionizing the field by enabling rapid and on-site diagnostic tests without the need for specialized laboratory equipment. Molecular POCT devices possess compact form factors, user-friendly interfaces, and leverage techniques like isothermal amplification and microfluidics, empowering immediate diagnosis and treatment decisions at the point of care. The transformative potential of POCT is particularly evident in resource-limited settings, remote locations, and emergency situations, greatly enhancing healthcare accessibility and improving patient outcomes. A noteworthy example of the application of molecular diagnostics in point-of-care testing is demonstrated in the research focusing on the development of diagnostic methods for African swine fever virus (ASFV) and classical swine fever virus (CSFV), which pose significant threats to the pig industry. The researchers introduced Recombinase-aided amplification (RAA) technology combined with a nucleic acid test strip (RAA-strip) to specifically detect ASFV/CSFV. This method exhibited high sensitivity and specificity, capable of detecting low levels of viral DNA/cDNA.

In the pursuit of medical precision, biomarkers have emerged as indispensable allies, providing measurable indicators of biological processes and conditions. Real-time PCR and microarray analysis have played pivotal roles in identifying and quantifying specific biomarkers. These advancements have propelled the development of companion diagnostic tests, revolutionizing disease diagnosis, prognosis, and treatment monitoring. By unravelling the intricate tapestry of biomarkers, molecular diagnostics empowers clinicians to identify patients who are most likely to benefit from targeted therapies, ultimately optimizing treatment outcomes.

In conclusion, the field of molecular diagnostics has undergone a breath-taking evolution, propelled by pioneering techniques, transformative breakthroughs, and cutting-edge applications. These advancements have reshaped healthcare, empowering clinicians with precise tools for disease detection, genetic analysis, and personalized treatment. As we embrace the ongoing advancements in this field, the future holds limitless possibilities for molecular diagnostics, promising further revolutions in healthcare and improved patient outcomes.

